# Effect of the *PPARG2* Pro12Ala Polymorphism on Associations of Physical Activity and Sedentary Time with Markers of Insulin Sensitivity in Those with an Elevated Risk of Type 2 Diabetes

**DOI:** 10.1371/journal.pone.0124062

**Published:** 2015-05-14

**Authors:** Thomas Yates, Melanie J. Davies, Joseph Henson, Charlotte Edwardson, David Webb, Danielle H. Bodicoat, M’Balu Webb, Philip Howard, Jackie A. Cooper, Steve E. Humphries, Kamlesh Khunti, Philippa Talmud

**Affiliations:** 1 Diabetes Research Centre, College of Medicine, Biological Sciences and Psychology, University of Leicester, Leicester, United Kingdom; 2 National Institute for Health Research (NIHR) Leicester-Loughborough Diet, Lifestyle, and Physical Activity Biomedical Research Unit (BRU), Leicester Diabetes Centre, Leicester, United Kingdom; 3 Leicester Diabetes Centre, University Hospitals of Leicester NHS Trust, Leicester, United Kingdom; 4 Centre for Cardiovascular Genetics, British Heart Foundation Laboratories, The Rayne Building, Institute of Cardiovascular Sciences, University College London, London, United Kingdom; 5 National Institute for Health Research (NIHR) Collaboration for Leadership in Applied Health Research and Care—East Midlands (CLAHRC—EM), Leicester Diabetes Centre, Leicester, United Kingdom; University of Catanzaro Magna Graecia, ITALY

## Abstract

**Background:**

Peroxisome proliferator-activated receptor gamma (PPARγ) is an important regulator of metabolic health and a common polymorphism in the PPAR-γ2 gene (*PPARG2*) may modify associations between lifestyle behaviour and health.

**Objective:**

To investigate whether the *PPARG2* Pro12Ala genotype modifies the associations of sedentary behaviour and moderate-to-vigorous intensity physical activity (MVPA) with common measures of insulin sensitivity.

**Methods:**

Participants with a high risk of impaired glucose regulation were recruited, United Kingdom, 2010-2011. Sedentary and MVPA time were objectively measured using accelerometers. Fasting and 2-hour post-challenge insulin and glucose were assessed; insulin sensitivity was calculated using Matsuda-ISI and HOMA-IS. DNA was extracted from whole blood. Linear regression examined associations of sedentary time and MVPA with insulin sensitivity and examined interactions by *PPARG2* Pro12Ala genotype.

**Results:**

541 subjects were included (average age = 65 years, female = 33%); 18% carried the Ala12 allele. Both sedentary time and MVPA were strongly associated with HOMA-IS and Matsuda-ISI after adjustment for age, sex, ethnicity, medication, smoking status and accelerometer wear time. After further adjustment for each other and BMI, only associations with Matsuda-ISI were maintained. Every 30 minute difference in sedentary time was inversely associated with a 4% (0, 8%; p = 0.043) difference in Matsuda-ISI, whereas every 30 minutes in MVPA was positively associated with a 13% (0, 26%; p = 0.048) difference. The association of MVPA with Matsuda-ISI was modified by genotype (p = 0.005) and only maintained in Ala12 allele carriers. Conversely, sedentary time was not modified by genotype and remained inversely associated with insulin sensitivity in Pro12 allele homozygotes.

**Conclusion:**

The association of MVPA with Matsuda-ISI was modified by *PPARG2* Pro12Ala genotype with significant associations only observed in the 18% of the population who carried the Ala12 allele, whereas associations with sedentary time were unaffected.

## Introduction

Peroxisome proliferator-activated receptor gamma (PPARγ) is a key regulator of fatty acid and glucose metabolism, adipocyte differentiation, and inflammatory processes. PPARγ exists in two isoforms: PPARγ1 and PPARγ2 [1]. PPAR-γ1 is expressed ubiquitously, whereas PPAR-γ2 is primarily expressed in adipose tissue. A common polymorphism in the PPAR-γ2 gene (*PPARG2)* alters the proline at position 12 to an alanine (Pro12Ala; rs1801282). This polymorphism is the only commonly occurring missense polymorphism in *PPARG2* in Caucasians. *In vitro*, Pro12Ala is a less active transcription factor, resulting in lower transcription levels of target genes [2]. The Pro12Ala variant is associated with a lower risk of type 2 diabetes in a dose-dependent manner, with a reduction in risk of 14% for each Ala12 allele carried [3]. Along with modifying the risk of type 2 diabetes, there is growing evidence showing that the Ala12 allele significantly modifies the relationship between lifestyle factors and metabolic health [4–7]. For example, intervention studies in the general population have shown that carriers of the Ala12 allele gain greater improvements to glucose and insulin metabolism following exercise training [4]. Pro12Ala has also been shown to modify the relationship between fat intake and metabolic health [5–7].

Whilst previous research investigating gene x environment interactions has focused on physical activity and dietary intake as exposures of interest, recent research has suggested that sedentary behaviour, defined as non-exercise sitting time, is also an important determinant of glucose regulation as well as morbidity and mortality outcomes independently of both obesity and MVPA [8,9]. Indeed, the inverse association between sedentary time and glucose regulation has been found to be particularly consistent in those with a high risk of, or diagnosed, type 2 diabetes [10–12]. However, the extent to which candidate genetic variants modify the relationship between sedentary behaviour and metabolic health in high risk subjects is unknown.

Given that PPARγ is directly linked to metabolic health, and considering the mounting evidence of a genotype x environment interaction, PPARG2 Pro12Ala represents one of the most promising polymorphism for further exploration of how specific genotypes modify responses or associations with different behavioural stimuli. This study aims to undertake a targeted investigation into whether the common Ala12 allele modifies the association between objectively measured MVPA, sedentary time and markers of insulin sensitivity in participants with an increased risk of type 2 diabetes recruited from primary care.

## Materials and Methods

### Participants

This study included baseline data from the Walking Away from Type 2 Diabetes study, the methods of which have been published elsewhere [13]. A total of 833 participants at an increased risk of type 2 diabetes were recruited from primary care, Leicestershire, UK, in 2010–2011; the present analysis was conducted in 2013. Individuals at high risk of impaired glucose regulation (IGR) (composite of impaired glucose tolerance (IGT) and/or impaired fasting glycaemia (IFG) and/or screen detected type 2 diabetes) were identified using a modified version of the computer based Leicester Risk Score, which was designed to be administered in primary care [14]. The Leicester Risk Score runs on primary care databases and ranks individuals for diabetes risk using predefined weighted variables (age, gender, BMI, family history of T2DM and use of antihypertensive medication). Those individuals scoring within the 90^th^ percentile in each practice were invited to take part in the study. This approach has been shown to have good sensitivity and specificity for identifying participants with an increased risk of IGR [14]. All individuals were unaware of their diabetes risk status before entering the study. Individuals were excluded if they had previously diagnosed type 2 diabetes or were currently taking steroids.

### Ethics statement

Ethical approval was obtained from the Nottingham Research Ethics Committee. Written informed consent was provided by all participants and measurements were performed by trained staff according to standard operating procedures.

### Sedentary and MVPA time assessment

At the baseline visit, all eligible participants were asked to wear a tri-axial accelerometer, (Actigraph GT3X, Pensacola, FL, USA), for a minimum of seven consecutive days during waking hours. Data were recorded in 15 second epochs. Previously used cut-points were used to categorise time spent in sedentary activities (<25 counts per 15 seconds) and time in MVPA (≥505 counts per 15 seconds) [15]. Non-wear time was defined as a minimum of 60 minutes of continuous zero counts and days with at least 600 minutes of wear time were considered valid [10]. In order to be included in the analysis, participants were required to have at least four days of valid accelerometer data [16]. A commercially available data analysis tool (KineSoft version 3.3.76, Kinesoft, New Brunswick, Canada; www.kinesoft.org) was used to process the accelerometer data.

### Demographic, anthropometric and biochemical measurements

Information on medication, ethnicity and smoking status was obtained following an interview-administered protocol conducted by a healthcare professional. Social deprivation was determined by assigning an Index of Multiple Deprivation (IMD) score to the participant’s resident area [17]. IMD scores are publically available continuous measures of compound social and material deprivation which are calculated using a variety of data including current income, employment status and previous education. Body weight (Tanita BC-418MA Scale, Tanita, West Drayton, UK) and waist circumference (midpoint between the lower costal margin and iliac crest) were measured to the nearest 0.1 kg and 0.5 cm respectively.

At baseline participants underwent an oral glucose tolerance test according to standard criteria. Participants were asked to fast from 10pm on the evening before the test and to avoid vigorous-intensity physical activity in the preceding 24 hours. Fasting and 2-hour post challenge (2-h) glucose samples were measured within the same laboratory within the Leicester Royal Infirmary, Leicestershire, UK, using a glucose oxidase method on the Beckman Auto Analyzer (Beckman, High Wycombe, UK). Plasma samples for fasting and 2-h insulin analysis were frozen within a −80°C freezer and analysed at the end of baseline data collection using an enzyme immuno-assay (80-INSHU-E01.1, E10.1 Alpco Diagnostics 26G Keewaydin Drive, Salem, NH 03079 USA). Insulin analysis was undertaken within a specialist laboratory by Unilever R&D, Bedfordshire, UK.

### Genetic analysis

Whole blood samples were collected during the baseline clinical assessment and stored at −80°C. Participants provided consent for the collection and storage of blood samples for genetic testing. Lack of consent for this aspect of the research did not preclude participation in the rest of the study. In total genetic samples were collected and analyzed for 620 (74%) of the Walking Away cohort. DNA was extracted using a standard protocol [18]; genotype was determined using the MetaboChip, a commercially available SNP chip, through methods described in detail elsewhere [19,20].Given the small sample size which prohibits multiple testing across numerous SNPs, this cross sectional study focuses on, and therefore only reports, the Pro12Ala (rs1801282) SNP in the *PPARG2* gene and can only therefore be considered as extending or confirming previous research rather than hypothesis generating.

### Statistical analysis

Insulin sensitivity was calculated using HOMA-IS [21] and Matsuda-ISI [22] according to the following formulas.

$$\text{HOMA}-\text{IS}=1/\text{HOMA}-\text{IR}\;=22.5/\left({\text{G}}_{0}\times {\text{I}}_{0}\right)$$

$$\text{Matsuda}-\text{ISI}\;=10000/\text{sqrt}\left({\text{G}}_{0}\times \;{\text{I}}_{0}\times \;{\text{G}}_{120}\times {\text{I}}_{120}\right)$$

These indexes of insulin sensitivity are commonly used in epidemiological research and have been shown to correlate reasonably with gold standard measures of insulin sensitivity and/or progression to type 2 diabetes [23,24]. By incorporating post challenge values of glucose and insulin, Matsuda-ISI is more likely to reflect factors related to insulin release and peripheral insulin resistance whereas HOMA-IS more closely reflects hepatic insulin resistance [25].

Forced entry linear-regression models were used to analyse associations between sedentary time and MVPA with HOMA-IS and Matsuda-ISI according to three models. Model 1 was adjusted for age, sex, ethnicity (White European vs. other), beta-blocker medication status, statin medication status, smoking status and accelerometer wear time. Model 2 was additionally mutually adjusted for sedentary time or MVPA. Model 3 was further adjusted for BMI in order to investigate whether adiposity attenuated observed associations. Interaction terms were fitted separately to Model 2 in order to assess genotype (Pro12 allele homozygotes vs. Ala12 allele carriers) x MVPA and genotype x sedentary time interactions. Significant interactions were followed by stratification.

HOMA-IS and Matsuda-ISI were logarithmically transformed to achieve normality; regression coefficients were therefore back transformed and represent the value by which the dependent variable is multiplied by for a given unit difference in sedentary time or MVPA. We display results per 30 minutes difference for ease of interpretation. In addition, results are presented as standardized regression coefficients to allow for meaningful comparisons across variables. P< 0.05 was considered significant for main effects and p < 0.1 was considered significant for interactions. All statistical analyses were conducted using IBM SPSS Statistics v20.0.

## Results

Genetic samples were collected and analyzed for 620 (74%) of the Walking Away cohort. Of these, 541 (65%) had valid accelerometer data and were included in this study. Those with missing data were younger (62.2 years for missing vs. 63.5 years complete; p = 0.023), had greater BMI (33.0 vs. 32.1kg/m^2^; p = 0.029), were more likely to be female (43% vs. 33%; p = 0.005) and were more likely to be from a Black and minority ethnic population (16 vs. 9%; p = 0.007). However, there was no difference in fasting or 2-h measures of insulin or glucose.

The number of Pro12 allele homozygotes in this cohort was 443 (82%), and the number with the Pro12Ala and Ala12Ala genotypes were 92 (17%) and 6 (1%) respectively. The genotype frequencies did not deviate from Hardy-Weinberg equilibrium predictions assessed by chi-square statistic (P = 0.62). The heterozygous and rare homozygous genotypes were combined for further analysis.

Table 1 displays participant characteristics stratified by genotype (Pro12 allele homozygotes vs. Ala12 allele carriers). Pro12 allele homozygotes had lower median levels of 2-hour insulin (43.9 vs. 60.7 mU/I; p = 0.022) and there was a trend towards lower levels of fasting insulin (8.7 vs. 9.8 mU/l; p = 0.065) and 2-hour glucose (5.9 vs. 6.6 mmol/l; p = 0.069). Insulin sensitivity (Matsuda-ISI) was also significantly greater in thePro12 allele homozygotes (5.3 vs. 3.7; p = 0.007). There was no difference between genotypes in levels of sedentary time and MVPA.

**Table 1:** Participant characteristics.

Variables	Ala12 carriers (n = 98)	Pro12 homozygotes (n = 443)	P value for difference
Age(years)	65 [59,69]	65 [60, 69]	0.485
Sex (female)	33 (34)	146 (33)	0.892
Ethnicity			
White European	89 (91)	401 (91)	0.927
South Asian	8 (8)	27 (6)	
Other	1 (1)	15 (3)	
Social Deprivation*	14.6 [8.3, 28.1]	12.5 [7.5, 21.6]	0.252
Beta-blocker medication	16 (16)	76 (17)	0.843
BMI (kg/m^2^)	30.9 [28.2, 34.5]	31.4 [28.4, 34.8]	0.776
Waist Circumference (cm)	99.0 [93.0, 107.5]	100.0 [93.0, 109.0]	0.654
Fasting glucose (mmol/l)	5.2 [4.9, 5.6]	5.2 [4.9, 5.6]	0.971
2-h glucose (mmol/l)	6.6 [5.1, 8.2]	5.9 [4.8, 7.8]	0.069
Fasting insulin (mU/l)^†^	9.8 [6.6, 13.8]	8.7 [5.9, 12.7]	0.065
2-h insulin(mU/l)^‡^	60.7 [31.7, 107.2]	43.9 [25.2, 75.3]	0.022
HOMA-IS	0.43 [0.29, 0.68]	0.50 [0.33, 0.76]	0.072
Matsuda-ISI^§^	3.7 [2.2, 7.2]	5.3 [3.2, 9.2]	0.007
Sedentary time (average mins/day)	623.7 [565.5, 687.7]	615.2 [549.7, 678.3]	0.304
Moderate to vigorous intensity physical activity (average mins/day)	33.0 [20.2, 50.8]	32.6 [19.3, 54.2]	0.845

Data displayed as median [IQR] or number (%)

* = higher values represent greater deprivation

^†^ = Fasting insulin data missing for 12 Ala12 carriers and 50 Pro12 homozygotes due to insufficient blood collection or cessation of bleeding

^‡^ = 2-h insulin data missing for 14 Ala12 carriers and 59 Pro12 homozygotes due to insufficient blood collection or cessation of bleeding

^§^ = Matsuda-ISI data missing for 21 Ala12 carriers and 89 Pro12 homozygotes; higher values indicate greater sensitivity

Table 2 details the results of the regression analysis for the overall sample. Both sedentary time and MVPA were associated with HOMA-IS and Matsuda-ISI. However, results for HOMA-IS were attenuated after further adjustment for each other (sedentary time or MVPA) and BMI. In contrast, associations of both sedentary time and MVPA with Matsuda-ISI were maintained across models. In the fully adjusted model, every additional 30 minutes spent sedentary were associated with a 4% (0, 8%; p = 0.043) lower Matsuda-ISI and every 30 minutes spent in MVPA were associated with a 13% (0, 26%; p = 0.048) higher Matsuda-ISI. The standardized regression coefficients for the above analyses are provided in a separate table (S1 Table).

**Table 2:** Associations of moderate-to-vigorous physical activity (MVPA) and sedentary time with markers of insulin sensitivity.

	HOMA-IS		Matsuda-ISI	
	β	P	β	p
**Model 1**				
MVPA	1.17 (1.09, 1.26)	<0.001	1.28(1.17, 1.40)	<0.001
Sedentary	0.94 (0.92, 0.97)	<0.001	0.91 (0.89, 0.94)	<0.001
**Model 2**				
MVPA	1.11 (1.01, 1.22)	0.040	1.17(1.03, 1.31)	0.012
Sedentary	0.97 (0.93, 1.00)	0.084	0.95 (0.91, 0.99)	0.017
**Model 3**				
MVPA	1.04 (0.95, 1.15)	0.267	1.13 (1.00, 1.26)	0.048
Sedentary	0.98 (0.95, 1.02)	0.351	0.96 (0.92, 1.00)	0.043

Coefficients represent the factor by which the measure of insulin sensitivity is multiplied by (95% Confidence Interval) for a 30 minute difference in sedentary time or MVPA

Model 1 adjusted for age, sex, ethnicity, smoking status, statin medication status, beta-blocker status and accelerometer wear time

Model 2 adjusted for above variables plus MVPA (for the sedentary time model) or sedentary time (for the MVPA model)

Model 3 adjusted for the above plus BMI

Significant genotype x MVPA interactions were observed for associations with Matsuda-ISI (p = 0.005), but not HOMA-IS (p = 0.890). There were no genotype x sedentary time interactions for HOMA-IS (p = 0.919) or Matsuda-ISI (p = 0.665). Table 3 shows associations of MVPA and sedentary time with Matsuda-ISI after stratification for genotype. MVPA was strongly associated with insulin sensitivity in Ala12 allele carriers; every additional 30 minutes spent in MVPA were associated with a 69% (16, 145%; p = 0.007) higher Matsuda-ISI. However in Pro12 allele homozygotes associations with MVPA were weaker, with every additional 30 minutes spent in MVPA only being associated with a 10% (−3, 24%; p = 0.113) higher Matsuda-ISI. Conversely, sedentary time remained inversely associated with insulin sensitivity in Pro12 allele homozygotes; every additional 30 minutes spent sedentary were associated with a 6% (1, 10%; p = 0.015) lower Matsuda-ISI. Thus only sedentary time was significantly associated with Matsuda-ISI in Pro12 allele homozygotes.

**Table 3:** Associations of moderate-to-vigorous physical activity (MVPA) and sedentary time with Matsuda-ISI after stratification byPro12Ala genotype.

	Ala12 carriers		Pro12 homozygotes	
	β	P	β	p
MVPA	1.69 (1.16, 2.45)	0.007	1.10 (0.97, 1.24)	0.113
Sedentary time	1.00 (0.88, 1.13)	0.994	0.94 (0.90, 0.99)	0.015

Coefficients represent the factor by which the measure of insulin sensitivity is multiplied by (95% Confidence Interval) for a 30 minute difference in sedentary time or MVPA

Adjusted for age, sex, ethnicity, smoking status, statin medication status, beta-blocker status, accelerometer wear time and MVPA (for the sedentary time model) or sedentary time (for the MVPA model)

## Discussion

This study found that the common *PPARG2* Pro12Ala polymorphism modified the associations of MVPA with insulin sensitivity (Matsuda-ISI), but did not modify associations with sedentary time. To our knowledge this is the first study to investigate the effect of the *PPARG2* Pro12Ala polymorphism on associations of objectively measured sedentary time and MVPA with measures of insulin sensitivity.

The strong modifying effect of the Pro12Ala polymorphism on associations of MVPA with insulin sensitivity is broadly consistent with previous epidemiological research using self-reported physical activity levels. Previous studies have consistently found a genotype x physical activity interaction, however the direction of the effect is equivocal; for example two studies have reported that the Ala12 allele in combination with low levels of physical activity conferred a particularly high risk of T2DM [26,27], whereas one study found that the combination of low physical activity and the Ala12 allele conferred a lower risk of impaired glucose tolerance [28]. In contrast, exercise training studies have been more consistent with the present study. To date five training studies have shown that Ala12 carriers have tended to be more responsive to the insulin sensitizing effects of exercise training compared to Pro12 allele homozygotes [29–33]. Our study extends these findings by suggesting this association is likely to be generalisable to a high risk population recruited from primary care.

Whilst evidence for the importance of the *PPARG2* Pro12Ala polymorphism on modifying the insulin sensitising effects of MVPA is growing, this is the first study to assess the impact on associations with sedentary behaviour. Sedentary behaviour has been hypothesised to be qualitatively different and independent to MVPA, both behaviourally and through effects on health [34,35].Our study adds to this research by suggesting that unlike MVPA, associations of sedentary time with insulin sensitivity are maintained in Pro12 allele homozygotes. Since Pro12 allele homozygotes represent at least 80% of the population [3], this result could help explain recent observational research which suggests that sedentary time may be a stronger determinant of metabolic health than MVPA in those at high risk of, or diagnosed, with type 2 diabetes [10–12].

The precise mechanisms underpinning the observations of this study are unknown. As *PPARG2* is primarily expressed in white adipose tissue, it is probable that any effect of the Pro12Ala polymorphism will be mediated through adipose tissue metabolism. It has been hypothesised that, as the Ala12 allele is likely to reduce transcriptional activity, it may enhance the ability of insulin to suppress lipolysis in adipocytes, resulting in the preferential utilization and oxidation of glucose in skeletal muscle which could be enhanced with physical activity [26].These potential mechanisms need further elucidation and our study suggests that they may not extend to sedentary behaviour.

The higher 2-hour insulin levels and lower insulin sensitivity seen in Ala12 allele carriers is in contrast to the majority of previous studies, where the risk of diabetes has been shown to decrease with each additional Ala12 allele [3]. However, our findings are consistent with those of the Finnish Diabetes Prevention Study which also included individuals with a high risk of diabetes and found that Ala12 allele carriers had higher levels of fasting glucose and a high risk of type 2 diabetes [26]. Furthermore, the Diabetes Prevention Program in the United States found that although the Ala12 was associated with a lower risk of T2DM, this positive effect disappeared in those with a BMI of 35 kg/m^2^ and higher [36]. Still other studies have shown that the Ala12 allele increases the risk of type 2 diabetes only when combined with low levels of physical activity [27]. Therefore the high risk nature of our cohort, with high levels of obesity and sedentary behaviour, may contribute to the baseline characteristics of our study.

Our study has several strengths and limitations. Strengths include the objective measurement of sedentary time and MVPA, which greatly reduces measurement error and variation compared to self-reported instruments, and that the Walking Away cohort was recruited from primary care and is broadly representative of individuals who are likely to be eligible for interventions aimed at the primary prevention of type 2 diabetes and cardiovascular disease. However, these cohort characteristics could also act as a limitation; the older age and elevated risk of type 2 diabetes may act to limit generalizability to the general population. Other limitations include the fact that genetic samples were not collected on the full cohort which may act to limit generalisability, however levels of glucose and insulin were similar in those with missing and complete data. Finally, the cross-sectional nature of the study design negates inferences of causality. For example, the effect of the Pro12Ala polymorphism on associations with sedentary time could be confounded by other unmeasured or imprecisely measured lifestyle behaviours, such as dietary intake. However, the strength of the modifying effect of the Pro12Ala polymorphism on the associations of MVPA, but not sedentary time, with a dynamic measure of insulin sensitivity, indicate the novelty of our findings and warrant further investigation by other epidemiological and experimental research platforms.

### Conclusion

Our study supports the importance of the *PPARG2* Pro12Ala polymorphism in modifying the beneficial effects MVPA, and furthers evidence by suggesting this effect is not extended to associations with sedentary time. The association of sedentary time with insulin sensitivity was evident in Pro12 allele homozygotes who represent the majority of the population whereas MVPA was only associated with insulin sensitivity in the 18% of the population who carried the Ala12 allele. If confirmed by intervention studies, this could have important implications for public health by suggesting that testing for the presence or absence of theAla12 allele could aid behavioural interventionalists in tailoring future chronic disease prevention programmes to optimise metabolic benefit. However, given the cross sectional nature of the study design and the other limitations, our results should not be interpreted as causal, but act as a stimulus for further experimental research.

## Supporting Information

S1 Table Associations of moderate-to-vigorous physical activity (MVPA) and sedentary time with markers of insulin sensitivity displayed as standardised regression coefficients. (DOCX)
