# Supplementary material for: Effect of the PPARG2 Pro12Ala Polymorphism on Associations of Physical Activity and Sedentary Time with Markers of Insulin Sensitivity in Those with an Elevated Risk of Type 2 Diabetes
Source: PLoS One. 2015 May 14;10(5):e0124062. doi: 10.1371/journal.pone.0124062 (PMC4431874; doi:10.1371/journal.pone.0124062)
Supplement: S1 Table — (DOCX) [file pone.0124062.s001.docx]

**S1 Table: Associations of moderate-to-vigorous physical activity (MVPA) and sedentary time with markers of insulin sensitivity displayed as standardised regression coefficients**

|  | **HOMA-IR** | | **Matsuda-ISI** | |
| --- | --- | --- | --- | --- |
|  | Standardised β | P | Standardised β | p |
| **Model 1** |  |  |  |  |
| MVPA | 0.20 (0.05) | <0.001 | 0.27 (0.05) | <0.001 |
| Sedentary | -0.25 (0.06) | <0.001 | -0.33 (0.06) | <0.001 |
|  |  |  |  |  |
| **Model 2** |  |  |  |  |
| MVPA | 0.13 (0.06) | 0.040 | 0.16 (0.06) | 0.012 |
| Sedentary | -0.14 (0.08) | 0.084 | -0.19 (0.08) | 0.017 |
|  |  |  |  |  |
| **Model 3** |  |  |  |  |
| MVPA | 0.06 (0.07) | 0.267 | 0.13 (0.07) | 0.048 |
| Sedentary | -0.08 (0.08) | 0.351 | -0.16 (0.08) | 0.043 |

Model 1 adjusted for age, sex, ethnicity, smoking status, statin medication status, beta-blocker status and accelerometer wear time

Model 2 adjusted for above variables plus MVPA (for the sedentary time model) or sedentary time (for the MVPA model)

Model 3 adjusted for the above plus BMI
